# Abstracts and Gleanings

**Published:** 1878-11-20

**Authors:** 


### *ABSTRACTS AND GLEANINGS.*

### STAMMERING.

A pamphlet, "Du Begaiment et de son Traitement Physiologique," by Dr. Jules Godard, although ostensibly written to bring into notice the method of treatment employed by a certain Professor Chervin, contains matter of some interest touching upon the medical history to this very common nervous trouble. It appears that Dupuytren was accustomed to recommend stammerers to adopt a method of speaking more like an operatic recitative than ordinary conversation. Itard, a French physician, devised a metal fork to be worn under the tongue. Rolier adopted the theory, highly satisfactory to the stammerers themselves, that their trouble was due merely to the fact that their vivid imaginations went too fast for the machinery which was to give utterance to their ideas. According to McCormac, the chief cause of stammering was the attempt to phonate with empty lungs, and the cure consisted in causing a deep inspiration to precede the act of speaking. Serres considered that to the respiratory trouble there was added the choreic inco-ordination of the muscles of articulation. Colombat adopted an elaborate classification of stammerers. Thus he divided them into two chief classes,—(*a*) the labio-choreic and (*b*) the gutturo-tetanic Tae labio-choreic admitted of four sub-classes,—viz., (1) deforming, grimacial, (3) aphonic (of women), (3) loquacious with spluttering, and (4) lingual with lisping. The gutturo-tetanic admitted six sub-classes,—thus: (1) dumb; (2) intermittent; (3) choreiform; (4) canine, in which the sound emitted resembles barking; (5) epiletiform, with excess of limb movement; and (6) idiotic. Hervey thought he saw the cause of stammering in the tongue being too short for the mouth, or the frænum too rigid. His treatment consisted in snipping the frænum or in wearing a metalic arc inside the lower dental arch, so that the tip of the tongue might be brought in contact therewith. Dieffenbach adopted the heroic measure of dividing certain of the muscles going to form the root of the tongue, and velpeau, following in the same direction, had recourse to the division of certain muscles, and Amussat also advocated similar severe surgical measures.

The classification adopted by Godard and Chervin is as follows: 1, inspiratory stammering; 2, expiratory stammering; and 3, mixed stammering; and each of these three classes is divided into a grimacial and a non-grimacial sub-class. The method of treatment adopted by Chervin is such as common sense dictates, and such as has been in ordinary use in this country. It consists in a systematic drilling of the muscles used in phonation and articulation, the pupil being made merely to imitate the movements of the master. The simple movements of respiration are first practiced rythmically and systematically, and if the patient be a very bad stemmerer, absolute silence is enjoined during the early days of his tuition. Then follow lessons on the simple vowel sounds, and then the rythmical utterance of words and sentences is adopted. With many stammerers the success of this method of treatment is considerable, and we doubt not that a large proportion may be practically cured by a patient drilling of this kind. Instances are tolerably common of stammerers who have broken themselves of their unfortunate habit by systematic drilling, and in extreme cases it might be necessary to follow the graduated system advocated by M. Chervin.—*London Lancet.*

### HYPODERMIC INJECTION OF ERGOT IN A CASE OF KELOID.

REPORTED BY DR. J. E. KEMPF.

The treatment of keloid tumors has so far been unsatisfactory. The following case is therefore of interest, as it illustrates the success of ergot in the treatment of keloid.

In the fall of 1876, Miss M., aged nineteen years, from Oil Creek, called upon my father, Dr. M. Kempf, to consult him about a growth on the right side of the face, over the lower jaw. It was a spontaneous growth of about two years' standing, resembling the cicatrix of a burn, perfectly smooth and solid, and accompanied by flashes of pain, lancinating in character. In the center of the growth the beat of a small artery could be felt. Dr. M. Kempf, not being certain whether the growth was malignant or not, advised an immediate removal, to which the patient consented. After the removal of the growth, nothing untoward occurring, the patient returned home.

In the spring of 1877, Miss M. called again upon my father, with a reappearance of the growth. It was now certain that the tumor was a keloid. Where the sutures had been inserted small keloids appeared. This, I believe, is characteristic.

Dr. Kempf could, of course, not expect any success from another removal; and so, after a consultation with Dr. Knapp, he advised the hypodermic injection of Squibb's ext. ergot, dissolved in alcohol. The patient's brother, being a physician, did the operation two or three times a week.

I heard nothing more of the case for at least a year, when, meeting the patient's brother again, he informed me that the keloid had entirely disappeared, after having been injected with ergot for several weeks.—*Lou. Med. News.*

### TREATMENT OF LEAD COLIC.

Dr. Kelly, in *New Preparations* says: In your last issue I see a note saying that a correspondent in Colarado wants some ideas on the treatment of "lead colic," or colica pictonum, as it is more scientifically termed. I have had quite a number of patients apply to me for relief from this very ugly disease, some of which were employees in the white lead works of this city. I have seen this disease in all its phases from the mildest type to the most violent, and the following treatment usually had the desired effect:

I first open the bowels in severe cases. I am as a rule compelled to resort to an injection composed of

Oleum ricini.
Molasses.
Saponis aa. ℥j.
Aqua Tepidæ Oj.
M.

This seldom fails, in fact it never has in my hands. I then relieve the terrible abdominal pain with opium and proceed to give pot. iod. in 25 grain doses 3 times per day. If the opium fails to afford relief I use chloroform by inhalation.

This treatment has never disappointed me, and I give it for what it is worth.

### CASCARA SAGRADO IN CONSTIPATION.

BY J. G. SUTTON M. D., GENEVA, OHIO.

In reading of cascara sagrado, which is so highly recommended through your journal, I concluded to try it in that much dreaded disease, constipation. Accordingly, I ordered some of Parke, Davis & Co. I tried it in a number of cases, in which it worked well, and in one case, which was especially interesting to me, it did more than I had expected. I was called to visit Mrs. S., who had been sick for the last three years, the last two of which she has not had a natural evacuation of the bowels, always being compelled to use an enema, which often failed to produce the desired effect, sometimes she would pass three or four days without an evacuation, although using an injection every day. She had taken cathartics without any benefit from a half dozen physicians from all schools, had adopted hygienic measures and carried them out well, but to no effect. I gave her

℞ Cascar sagrado, ℥j.

Berberis aquifolium, ℥j.

Syr. simplex, ℥ij.

and ordered her to take a teaspoonful four times a day until her bowels acted freely (as I had forgotton to state, her bowels had not moved for four days when I first began its use), then but three times a day; the desired effect was soon produced. The dose was diminished one teaspoonful per day, and before she had taken the second prescription she said she needed no more medicine, and now she appears natural in that respect.—*New Preparations.*

### TRANSLATIONS.

### CURE OF ONYCHIA MALIGNA.

Dr. Gaetano (*Jour. des Sci. Med.*, 1878, p. 463; from *Il Morgagni*) was called to a little girl 10 years of age who had suffered horribly for six months with peri- and subungual ulceration of the right index finger. Having softened and raised up the nail as much as possible, he dropped a concentrated solution of morphia upon the sore, with which he kept it in contact a quarter of an hour. He then covered the diseased part with very finely powdered nitrate of lead, and enveloped the finger with a bandage. The pain was almost immediately relieved, and the patient slept soundly that night for the first time in a long period. The bandage was removed at the end of five days, and the ulcer was found about one-half healed. The application was repeated, and at the end of five days more complete cicatrization had taken place. In two other cases results as favorable were obtained by Dr. Gaetano, the applications being made more or less frequently as the case seemed to demand.—*Medical Times.*

---

### SPINAL DISEASE TREATED BY SAYRE'S PLASTER JACKET.

---

Dr. Mac. Naughton, in *British Med., Record,* said: "We are all too prone to run after new forms of wonderful cures; and the cruical tests which time alone supplies are overlooked or ignored in our desire to applaud an invention, or see in it some advantage over other menns which may have failed to satisfy our wishes, or which do not meet all the difficulties which a variety of cases is sure to present. He did not think it right to expect more than average results from any treasment. Cases must constantly occur which fail under any treatment; and it must have happened in the case of the plaster jacket, that it has been applied in instances where no permanent cuie, nor indeed temporary benfit, could be affecthd, so advanced the disease, and so great the deformity. Parents have been so anxious and solicitous to try the benefit of the treatment, that he was sure many cases were put up where no material good or benefit with justice, could be looked for. He had traced the results in the cases now on record, to the past month. Tho majority of them had been under treatment for a period of qeteen nine and ten months. As yet it was impossible to state decidedly permanent results in the majority of the cases; but, from the details of those read, it would be seen that the superiority and benefit of the jacket over other means of treatwent was unquestionable. A large number of the cases quoted had been treated by other appliances made by the best makers, and under surgical advice; little or no improvement had taken place, notwithstanding that these supports had been worn for months or years; while the advantage of the jacket was immediate. Some of patients whose history was read by Dr. Jones, and who are now walking about, restored to health under this treatment, had been confined to bed, or kept in the horizontal posture for years. In only a few cases of the entire of those taeated by him had there been what he would call, up to the present, failure.

---

### GENITO-URINARY SURGERY.

---

Mr. Reginald Harrison, of Liverpool, spoke in very favorable terms of the apparatus devised by Dr. Bigelow, of Boston, U. S., for use in litholapaxy, and his method of treating stone in the bladder. Mr. Harrisson had recently, during a visit co the United States, turough the kindness of Dr. Bigelow, seen this operation practed. Though the number of cases was at present few, yet the results have been satisfactory as to demand for Dr. Bigelows proposals the very serious attention of those interested in the subject. Mr. Harrison considered that, whether or not we snould be prepared to accept in their entireity Dr. Bigelow's proposals, the adoption of improved means of evacuation would tend to diminish the rate of mortality following lithotrity.

Dr. W. H. Pancoast, of Philadelphia, showed drawings of an instrument modified on the plan of Syme's urethrotome, which he was in the habit of using in operating on sttricture of the urethra. He had used it in fourteen cases, some being of a severe character. The instrument conceals a knife, which can be pushed forward by a screw, and is guided by a whale-bone bougie.

Dr. J. P. H. Boileau urged the treatment of syphilis without mercury. He employed hot water baths, confined to bed, scrupulous cleanliness, a full diet, iodide, chlorate and nitrate of potassium, Dover's powder, etc. His experience was questioned by most of his hearers.

### OIL OF AMBER IN ANGINOSE AFFECTIONS.

For all that is known or said of it, rectified oil of amber might as well have been retired long ago to that silent majority, the non-officinals; but I hope to suggest a use for it that may revive, at least, a measure of its ancient praises.

I should preface my clinical detail, however, by stating that the remedy has been put upon trial by me for a number of years in many cases, diverse in age, habits, circumstances, in short, in their entire environments.

It must also be understood that in several of the cases here described the oil of amber was merely adjuvant to the general treatment of the patient's disease, and that it was expected to do only one thing—to relieve the cardiac pain.

The question of organic or merely functional disease is not considered, as the amber is recommended only for the neuralgic element, no matter how associated or excited, except, however, that the medicine is a stimulant, and is not thought appropriate in sthenic cases and cases of "active aneurism" or ventricular dilatation with much hypertrophy.

Some years ago, when I lived in Wheeling, the brothers Dr. Cummins invited me to see an old lady who had suffered for years from sharp stitches in her heart, which grew in violence and frequency with her age, and her bowels were habitually costive. Her physicians had failed to find an effective means against either trouble. I proposed she should take from four to six, and, if need be, from eight to twelve, drops of rectified oil of amber on a lump of sugar, melted in water, and repeat the dose every thirty to forty minutes at each paroxysm until better. So prompt was her relief each time that the agency of the amber could not have been mistaken; it also usually acted on her bowels.—*Dr. Finck, in Med. Times.*

### THE INFLUENCE OF SALICYLATE OF SODIUM IN THE TREATMENT OF DIABETES.

Dr. Miller, ot Kiel (*Bull. Gen. de Therap.*, 1878, vii. p. 142,) concludes from his experience that salicylate of sodium causes the temporary disappearance of sugar from the urine, the improvement being more rapid under large doses, where these are tolerated by the patient. The average dose at first is nine to ten grammes daily (one hundred and forty to one hundred and fifty grains,) but, as the effect of the salicylate diminishes rapidly, the dose must be increased to fourteen or sixteen grammes daily, in order to hope for continued improvement on the part of the patient. Salicylate of sodium can be administered in large doses and for a long time without danger. Should toxic symptoms show themselves, these will rapidly disappear on the cessation of the medicine.—*Med. Times.*

TO CONCEAL THE TASTE OF QUINIA.—Dr. S. Ashhurst says that if cinchona be mixed in the proportion of one grain of the alkaloid to four grains of sugar of milk, and one-tenth of a grain of bicarbonate of sodium, it will leave no bitter taste in the mouth. The mixture may be taken dry or dissolved in water.—*Druggists' Circular.*

HICCOUGH CURED BY COMPRESSION.—A case is cited from a French journal, in which hiccough which had been "incessant for fifty days" was cured in five minutes by powerful compression over the epigastrium. All other conceivable means had failed.—*Pacific Mediea and Surgica Journa,* August, 1878.

### VALENTINE'S MEAT-JUICE.

This preparation, as its name implies, is the juice of meat expressed by a powerful hydraulic press. Unlike simple extracts of meat it contains a large quantity of albumen in solution along with some hæmoglobin. When heated, or when mixed with hot water, the albumen is precipitated, and the hæmoglobin coagulated, so that the reddish colour of the solution is changed to a light brown, and the liquid becomes turbid. The makers, therefore, recommend that it should be mixed with cold or moderately warm water. We find, however, that when boiled or mixed with hot water, it forms a very palatable beef-tea, and in many cases might be given in this form, although in cases of great exhaustion, where the utmost nutriment is required, the mixture with cold water might be better. The makers also recommend that admixture with acids be avoided; but it has been shown that the imperfect digestion in fevers depends on want of acid in the stomach, and not on want of pepsin. The addition of a little dilute hydrochloric acid to meat-juice and water makes a very pleasant drink, and likely to be grateful to patients. Some of the albumen of the juice may be absorbed from the stomach unchanged, but part will probably be digested, and the acid will hasten this process. Altogether we think that this juice will be of great service to the physicians as affording a concentrated and easily assimilated form of nutriment.—*The Practitioner,* June 1878.

### COTO BARK.

Coto was first employed clinically (in Germany) by Dr. Gietel in Munich.

He used both the powdered bark and the tincture, one part of bark to nine of 85 per cent. alcohol. Gietel concluded that coto is a specific in diarrhœa. Burkart and Riecker, experimenting also with cotoin, came to the same conclusion as Gietel. Since February, 1877, Fronmuller himself employed coto in some two hundred cases of various diseases, of which ninety-three suffered with colliquative diarrhœa, and ninety-one from sweating of the same grave character. Of the various preparations employed, tinctura cotonis was used in one hundred and nine cases in the dose of about one hundred drops per day on an average. In twenty-four cases cotoin was used in the dose of 0. 1. to 0. 3 decigramme several times a day in powder. In five cases paracotoin was administered in somewhat larger doses; in five cases soft coto resin to the amount of 0.12 to 0.6 decigramme per day in pill form. The medicine was usually directed against the excessive diarrhœa or sweating; in a few instances against the loss of appetite. In ninety-two cases of colliquative diarrhœa occurring in typhoid fever and tuberculosis, fifty were entirely relieved, twenty-six were improved, and nine remained unaffected. In some cases the diarrhœa relapsed, but was again checked. In most cases, however, good stools were soon obtained. The best results were gained by large doses. The tinctura cotonis was given in water, and taken without difficulty. In a few cases the patient complained of burning and tickling in the throat; this appeared due to some want of care in preparation. Coto has the advantage over other similar remedies used in diarrhœa, that it *improves* the appetite. A medium dose of the tincture is fifty drops, thrice daily, given on sugar or in water.—*Medical Times*

### AILANTHUS GLANDULOSA.

The "tree of heaven," as it is called, which in its home attains a height of 80 to 100 feet, is used in Greece as an ornamental roadside tree, but during the period of flowering it gives oft very disagreeable odors, whence it might more aptly be called "stink-tree." In this respect it resembles the stinking-bean trefoil, *Anagyris fœtida.* Many years ago the bark of Ailanthus was praised as a remedy for tapeworm. My own experiments convinced me of its usefulness, at least in dogs, to whom I administered a saturated decoction of the fresh bark. Hunting dogs, imported from western Europe into Greece, are very apt to be affected with tapeworm, when they rapidly become emaciated and die, unless speedily relieved.—*New Remedies.*

### STRYCHNIA OR NUX VOMICA AS A TONIC.

In the *Practitioner,* Dr. T. Lauder Brunton says strychnia is at once a gastric, vascular and nervous tonic. It has, with the exception of quinine, a more powerful action than most other bitters, in preventing putrefaction. It excites the sensibility of the vaso-motor centre, thus exerting a beneficial effect upon the circulation, and likewise directly stimulates the nervous tissue of the spinal cord itself. So great is its effect upon the vaso-motor centre that by its means physiologists have discovered that instead of being confined to the medulla oblongata, as was formerly imagined, this centre extends down the spinal cord. It has just been said that an impression made upon the sensory nerves reflexly stimulates the vaso-motor centre, contracting the vessels and raising the blood-pressure, but when a cut is made across the spinal cord, just below the medulla oblongata, this result is not produced. From this experiment it has been concluded that the vaso-motor centre was entirely confined to the medulla oblongata above the place of section; but if a little strychnia be now injected into the veins of an animal in which the cord has been thus divided, and a sensory nerve be then irritated, the vessels will contract and the pressure of the blood will rise. It thus became evident that the vaso-motor centre extends down the cord from the medulla, but that its spinal portion is so feebly developed that under ordinary circumstances it has no power to contract the vessels when reflexly excited by stimulation of the sensory nerve. But strychnia has the power to increase its excitability so much, that reflex stimulation in this way will produce through it a decided effect. Now, when we consider that sensory impulses are proceeding every moment from the skin to the vaso-motor centre, we can readily perceive how a slight increase in susceptibility, produced by strychnia, will have a wonderful effect in raising the tone of the vessels, and aiding the circulation. The mode in which quinine acts is not so clear, but we know from observation, that, also in small doses, it renders the pulse stronger and less compressible.

From what has just been said, then, it would appear almost that strychnia or nux vomica is one of the most valuable tonics which we possess. When combined with nitro-hydrochloric acid, it is perhaps, one of the most efficient remedies that we can give for the debility which is so often noticed in warm weather, and when the ordinary tonics, such as gentian, columbo, cascarilla, or quinine do not produce the desired results, the addition of a little nux vomica or strychnia to them may give us the wished-for effect.—*Medical and Surgical Journal.*

### CONTRACTION OF THE FINGERS.

(*The British Medical Journal,* June 29, 1878).—Mr. William Adams thus describes the operation and treatment which he now practices in cases of Dupuytren's contraction:

1. The subcutaneous division of all the contracted bands of fascia which can be felt; the bands to be divided by several punctures, with the smallest tenotomy-knife passed under the skin and cutting from above downwards, a pledget of lint being at once placed over each puncture and retained in position by a strip of plaster.

2. Immediate extension to the full extent required for the complete straightening of the fingers, where this is possible, and the application of a retentive well-padded metal splint from the wrist along the palm of the hand and the fingers; the fingers and hand to be bandaged to the splint.

3. The bandage not to be removed until the fourth day, when the lint and plaster may also be taken off, as the cutaneous punctures are always found to be healed by the fourth day. The retentive metal splint to be re-applied, and the hand and fingers bandaged to it.

4. Extension to be kept up by the splint worn continuously night and day for two or three weeks; but the splint and bandage to be changed every two or three days. After this the extension splint is to be worn at night only for an additional three or four weeks, free motion being encouraged during the day.

### TREATMENT OF GLANDULAR SORE THROAT.

(*The British Med. Jour.* July 6, 1878). —Dr. James Sawyer writes as follows of his treatment of this disease: Glandular sore throat, by which is meant catarrhal congestion or inflamation in and around the glandulæ of the mucus membrane of the pharynx and larynx, is a very tedious and troublesome affection. It has been known as dysphonia clericorum; it is, in fact, the chronic sore throat to which persons are liable who use their voices extensively, especially in large rooms or in the open air. Dr. Sawyer desires to draw attention to the usefulness of the topical application of borax in its treatment. He orders a saturated aqueous solution, which the patient applies to his throat by the aid of Corbyn's throat-spray. The spray should be employed for several minutes thrice or more frequently daily; and midway between meals. If the larynx be much implicated, the patient should inspire deeply while the spray is playing upon his throat. This very simple method of treatment has lately been found to be of striking service. The cure may be expedited by the application of astringent solutions to the pharynx and larynx by means of suitable brushes. When there is much secretion, extract of eucalyptus is a good local astringent, which may be used in the form of lozenge.—*Ib.*

### DEATH FROM ERGOT EMPLOYED TO CAUSE ABORTION.

J. C. Lincoln, M. D., of Bowling Green, Ohio, reports a case of the above-mentioned nature, in which the effects produced by the drug were swelling and discoloration of the lips and face, dilated pupils, anxious and alarmed expression of countenance, pulse of forty-eight and weak, cold extremities; purplish hue of skin over entire surface, persistent vomiting and purging, and an alarming degree of prostration. Coma supervened; action of heart became more feeble, breathing irregular, and death took place two days after the abortion occurred, and four days after taking the ergot, which was afterwards ascertained to have been in the form of fluid extract, and in quantity four ounces.—*New Remedies.*

### MANAGEMENT OF THE BREASTS WHERE NURSING IS NOT ADOPTED.

Nearly all the speakers agreed (at New York Academy of Medicine) that the best plan was to let the breasts entirely alone. Dr. Hubbard said that such had been his practice during his connection with the Infant Asylum, where most of the mothers did not nurse their children, and during the whole of that period there was not a mammary abscess formed. The pain in the breasts subsided, as a rule, within twenty-four or forty-eight hours, and no further trouble was experienced if no attempt was made to draw the milk. If the milk was drawn only once, the character of the case was entirely changed. This method of treatment appears to us quite rational, and if at the same time the bowels be kept freely open and the diet restricted, it will generally be successful.

---

LOCAL ANAESTHETIC.—As a local anæsthetic to apply to gums, in the extraction of teeth we find the following (*in Dental Cosmos.*)

℞. Pulo Camphor........................dr iv
Sulphuric Ether........................oz i

Apply to the gum around the tooth with a pledget of cotton moistened with the mixture, until the gums turn white, when the tooth can be extracted with little pain.

---

*Iodoform Ointment.*—Iodoform 5 parts; hog's lard, 45 parts; to be melted together at the temperature of a water-bath, and stirred until cool. To be marked "for external use." In pruritus, prurigo, chronic eczema, fissures, and painful ulcers.—*Lancet and Clinic.*

---

### HOT MUSTARD BATHS IN PNEUMONIA OF CHILDREN.

Dr. Weber in American Journal, obstetrics reporting a case says:

I recognized pneumonic infiltration of both upper lobes. In spite of emetics, digitalis, mustard plasters and poultices over the chest, she became cyanotic at the end of the third day, with stertorous breathing, cold extremities, and failing heart's action. It occurred to me at this stage to immerse the patient in a hot mustard bath of 105° F.. prepared by diffusing about a pound of mustard in a baby tubfull of hot water. I kept her in for about ten minutes, making thorough friction all over the surface, and until the skin had assumed a pinkish color. After being put to bed, which I had well warmed previously, the child began breathing easier and soon fell asleep.—The skin remained warm, and an hour after the bath the child was perspiring freely. With the improvement of respiration, the pulse became stronger and less frequent, and the child took the breast readily. Encouraged by this success, I repeated the process four hours later with the same good result; and after having administered five baths in the course of forty-eight hours, and given no medicince whatever, I had the satisfaction of seeing my patient convalescent."

---

### CALOMEL.

---

Dr. McElroy, in a report to the *Gainsville Ac. of Med.* says:

"So calomel has held its place in therapeutics for centuries; its use having been at times violently opposed, even to calling into existence sects in medicine, founded only on the single idea of not using it at all. Perhaps the abuse of it was chief reason for the birth of homeopathy, and its life—a vigorous one in many centres of population—in our day.

I have somehow got to giving much more credit and importance now, than formerly, to the Latin maxim 'Vox Dei,' or possibly its fellow, "Vox Populi, Vox justiitiæ," *i. e.* that the ultimate judgments of the people are always just.

The people seeing the injurous effects of calomel, as used by the profession, rebel against it. The profession is firm in its position. New sects are called into existence to oppose its use. When the profession learns the lesson the people, designed to teach, these sects pass out of existence. The people are, after all, the ultimate arbiters in regard to medical practice. If patronage is withdrawn from the regulars, or from any sect, they, or it, must soon pass out of existence.

For some years during my professional life, I think I did not prescribe ten grains of calomel per year. And I could not get along without it if people would have patience without substitutes. But they did not like to take alkalies long enough, nor wait the effects of podophylln, iodine, etc., nor would they have patience. But every now and then one would leave me. So, in self-defense, possibly self-preservation, I had to resume its use. But I had become, as I believe, wiser during the period of non-using. It is, to say the least, a wonderful effecacious agent in the hands of actual working practioners; and as happily convenient. The ideals now in use in the profession regarding its *modus operandi* are purely scholastic, having little or no representation in nature. Cathartic, chologogue, alterative, deobstruent, etc., are fictions of men. Calomel, as all "stored up" force in the same material, in the same chemical condition, but has one effect, increased or decreased according to quantity, and time, in its introduction into living bodies. It is material, so far as the mental mercury is concerned—not a natural constituent of living bodies. Its operation is to quicken the pace of the waste of existing tissue. The growth of living bodies has limitations. Flesh, living flesh, which does not disintegrate, performs no natural function. Where such an event takes place in a living body, the result is called a "tumor," "morbid growth" or hyperthrophy." The stoppage of physiological decay of living tissue is death. The retardation of physiological decay constitutes a pahthological condition. The pathological condition, so to speak, ceases to be such, when the pace of molecular disintegration is hurried up, no matter by what means, to the physiological velocity. I do not, therefore, now associate with its administration conception of "unloading the *primae viae*', "rousing up the secretions," or stirring up the "bile." My concepts are limited to quickening that pace of molecular works, not alone in the abdomen but thoughout the remotest parts of a living body.

I know now that my mistake in early professional life was in too large doses, giving too much at a time, repeating too often. In its employment now, with the better understanding of its effect, I rarely ever give more than half a grain, and not often nearer together than six hours, if the dose is to be repeated. In the bulk of cases the bowels will operate freely inside of twenty four hours. A failure to do so indicates some modifications of structure in the nerve masses, or muscular structure, *i. e.* partial paralysis, so to speak. Singularly enough I find one of its highest uses to be in delicate and weakly persons, with whom tonics disagree, or fail to work satisfactorily, or to be followed by improvement. Half a dozen granules of half a grain each at from six to twelve hours' interval, seem to get up the requisite speed in molecular changes, followed by the culmination of effect, or dead matter; when tonics will be followed by the desired results, unless there are permanent changes of structure to modify, or wholly prevent them.—*Cin. Md. News.*

### GLYCEROLE OF SUBACETATE OF LEAD IN ECZEMA.

CASES BY PROF. DUHRING.

For an account of the theory of action of glycerole, reference may be made to Mr. Squire's papers. The formula which he suggests is as follows:

Acetate of lead, 5 parts;
Litharge, $3\frac{1}{2}$ parts;
Glycerine, 20 parts, by weight.

Mix, and expose for some time to a temperature of 350° F. Filter through a hot water funnel. The clear viscid fluid resultant contains 120 grains of the subacetate of lead to the ounce. It is used as a stock from which the preparations employed are made by dilution with simple glycerine. In the cases below noted, the strength of the glycerole in any given instance is designated by the number of grains of subacetate of lead which it contains.

Although, as the title of this paper indicates, the majority of the cases of which notes are here given belong to the category of eczema rubrum, yet some instances are included of other forms of eczema. These are introduced with a view to show the effect of the glycerole in these cases, and for purposes of comparison.

Case I.—Myra W., æt. 55. Applied for treatment January 18. *E. rubrum, leg.* Disease covers the limb from ankle half-way to knee. There is œdema, infiltration, a red, weeping surface, varicose veins, intense itching and burning. The patient a large, stout woman; the affection of some months' duration. Glycerole gr. xv. ad ℥i. Bits of rag saturated with lotion to be laid freshly on affected parts night and morning, and then bandage to be applied. Limb not to be washed excepting when crusts accumulate, then with hot water and brown soap. Jan. 23.—Improvement. Less œdema, little weeping, no itching or burning. The leg had only been washed once, on account of the pain this procedure caused. There was a thin white deposite over the surface. Jan. 30.—The œdema and infiltration have become so rapidly reduced that the skin is wrinkled. Pigmentation beginning. Color of surface dusky red, inclining to brown. Patient delighted with success of treatment. June 20.—No medicine for a month or more. Completely cured, excepting that a small patch of infiltrated skin remains on instep.

This was in every way a typical case of eczema rubrum, and exemplifies very well that form of disease in which the glycerole appears to act to the best advantage. The rapid diminution in the œdema and swelling was striking, and the relief given to the patient was remarkable.

Case II.—Mary Ann S., æt. 47. Applied for treatment May 7. *E. rubrum, leg.* Palm sized patch over front of leg, including a shallow ulcer the size of a quarter dollar. The diseased skin red, nifiltrated, and slightly moist. Sensations both of itching and burning. The limb swollen and œdematous, and showing varicose veins. Some months' duration. Glycerole gr. xx ad ℥i. Not to be washed. Elastic stocking. May 16.—Great relief. May 24.—Patch of eczema one-half original size. Ulcer entirely healed. June 6.—Nearly well.

The rapidity of cure in this case was somewhat unusual for a patient obliged to pursue her ordinary avocations without cessation. It may be attributed in part to the aid given by the elastic stocking, but we are quite sure that this alone would not have cured a patch of chronic eczema with ulceration unless the glycerole had also been carefully and thoroughly applied.

Case III.—Christiana D., æt. 68. Applied March 19. *E. erythematosum et fissum, palms and soles.* Aggravated case. Palms show a not unusual degree of the disease. Soles very severely affected, covered with tough, greenish-brown, heaped-up masses of horny epidermis, and deeply fissured, looking like rhinoceros hide. Least movement causes skin to crack, and gives intense pain. March 26.—Glycerole gr. xv. ad ℥i. April 2.—Improvement. Skin less red; fissures closed; much less scaliness. Soak the feet every evening in hot water, and wash them with brown soap before applying the glycerole. May 8.—Palms nearly well; only a few small scaly patches remain. Backs of hands itch. Wash with tar soap. Soles much improved, though still scaly.

Though not belonging to the class of E. rubrum, this case is noteworthy as showing the good effect of the glycerole in a peculiar intractable form of eczema, where, it should be said, other forms of internal and external treatment had failed.

Case IV.—Jane McC., æt. 42. Applied February 5. *E. squamosum et rubrun, leg.* Hand-sized patch over front of left ankle and instep; similar but smaller patch on right sole. A number of varicose veins about ankle. Glycerole gr. xx. ad. ℥i. Leg not to be washed, but to be bandaged with great care. Feb. 7.—Improvement. Tendency to the formation of pustules here and there, both over seat of disease and in neighborhood. Feb. 12.—Nearly well. Thick deposits over skin. Wash off. She came two or three times more, always improving, and was discharged, finally, quite well, April 4.

A very satisfactory result. The affection was practically cured in a week, though it took some time longer to remove all traces of its existence. The formation of pustules in this case did not interfere with the cure. Usually, the appearance of these lesions signifies that the glycerole does not agree.—"Medical Times."

### ON THE USE OF WINE IN THE CAPILLARY BRONCHITIS OF CHILDREN.

M. Bonamy, of Nantes speakes highly the use of wine in the treatment of pillary bronchitis in very young children. He does not think very large doses advlsable; he does not think very large doses not think very large doses advisable; he gives from five to siz ounces daily to children between one and two years of age, varying the dose also according to the strength of the patient. As adjuvants he employs cutaneous revulsives and ipecac. He reports three cases in which this treatment was crowned with complete success. One was a case of severe broncho-pneumonia; from five to six ounces of Malaga wine were given daily, and under its influence the respiration fell rapidly from 50 to 26 in a minute. In the other two cases he had to deal with broncho-pneumonia complicating pertussis; one of the patients was in a state of collapse when the treatment was begun. He used the following formula in these cases: Malaga wine, ℥ iij.; peppermint water, ʒ ijss.; syrup of orange-peel, ʒ v. M. A dessertspoonful every hour.—*Jour. de M. d. et de Chir.*

### NEUTRAL AGETATE OF LEAD IN GRANULAR CONJUNCTIVITIS.

Dr. Pierd'houy employes finely powdered, neutral acetate of lead in the following manner, in the treatment of conjunctival granulations: a blunt-pointed hair pencil is slightly moistened, impregnated with a small quantity of the powder, and then brushed lightly over the exposed surface, so as to touch all the greuulations. The mucosa must first be carefully cleansed. He claims thet incrustations on the cornea are not to be feared. even when ulcers exist. The granulations, after the application, appear flattened and shrivelled, and are covered with a delicate, whitish pellicle. the reaction does not last longer than twenty-four hours, but it is sufficient to repeat the operation every four or five days. The advantages claimed for this treatment over that by nitrate of silver or bluestone are that it is less painful, that cicatrices are less likely to form, and that the time revuired to produce atrophy of the granulations is only about half as long.—*Le Lyon Medical.*

### TREATMENT OF CROUP BY INJECTION OF LIQ. FERRI SESQUICHLORID INTO THE LARYNX AND TRACHEA.

Dr. Palvadeau in the Union Menicale 1878, No. 86, reports two cases of croup that were treated by him and Regi by means of injections of liquor feri sesquichlorid that reeovered. His method is as follows: "15 drops of the liquor are mixed with 15 drops of water and this solution is injected by means of a hypodermic syrings into larynx. The needle of the syringe is introduced to the depth of 1—1½ centimeters, above the thyroid cartilage and then 5 to 6 drops of the solution are then injected. Two hours after the injection has been made an emitic is adminiotered. Uusually the membranes were expectorated after a short time. If improvement does not follow in a short time the injection must be repeated." Regi, in his case was forced to inject several times; finally, however, membraues were spit up. Palvadeau considers this method entirely devoid of danger and producing no harm.—*Lancet and Clinic.*

### THE COLLODION BANDAGE IN THE TREATMENT OF UMBILICAL HERNIA.

In the Vienna general Policlinic, the collodion bandage of Rapa (of Naples) is used. It is applied in the following manner: The mother takes the child on oer lap, the shoulders lying on the left, the hips on the right leg. The upper extremities of the child are held fast by the left hand of the mother, the lower extremeties by the right hand.

The hernia and its visinity are now penciled over with a broad layer of collodion. The hernia is now reduced and a folded compress 4 centimeters wide and 3 centimetres long is placed over the ring, the side next to the hernia having been covered with collodion. This compress is held in place by an assistant, a long strip of adhesive plaster, 3 centimeters broad, is placed over it. This strip must be long enough to pass around the body and cross upon the abdomen. During the application of the plaster the recti-muscles must be pressed together by an assistant. Finally, over this a linen bandage equally long anc broad is applied, and the entire surface of the bandage over the abdomen is covered with collodin.

To protect from eczema, Monti applies a mivture of emplast. diachyli simp. and cerat. fuscum, instead of the adhesive plaster. The formula is, emplast. diachyl. simp.,30; cerati fusci 10; ol. oliv. 9.5; ut liquifact, ft. emplast.—*Lancett.*

### REMARKS ON EXTRACT OF MALT AND ITS COMBINATIONS.

From a paper read by J. J. Mulheron, M. D., at a meeting of the Wayne County Medical Society.

I appreciate the fact that many of our more conservative members are inclined to look with distrust on many of the new preparations which enterprising pharmacists are giving to the profession. This feeling, it must be admitted, has too much foundation in the impositions which unscrupulous manufacturers have practiced; but there is a possibility of carrying it too far. The profession of this country, besides being intensely practical, is also tolerably discriminative; it soon learns what estimate to place on anything it has put to practical test, and the fact that an article is spoken of with favor some years after its introduction is pretty good presumptive evidence of merit. This time-test successfully withstood, should justify a trial of any remedy by the most conservative. * * * Among articles of comparatively recent introduction in this country may be mentioned Extract of Malt, prepared after the method of Trommer. Although for a long time enjoying the confidence of the profession of Europe, and particularly of germany, it has been before the profession of this country for only about five years. It has stood the time-test alluded to, and is to-day more popular than at any time since its introduction. Its combinations with other articles has increased its popularity, and these combinations are, perhaps, more generally employed than either of their constituents.

My experience with Extract of Malt has been confined to its use in diseases of mal-nutrition and mal-assimilation. In such affections the stomach and the digestive portion of the intestines are primarily involved, and these failing in their functions, decomposition takes the place of digestion and we have absorption of unnatural products, with the long line of disorders which follows, and to which a variety of names is applied.

Before any local trouble manifests itself dyspeptic symptoms call for relief, and the failure in the digestion of carbonaceous ingesta is rapidly followed by loss of weight, sometimes even to emaciation. Theoretically, then, the remedy indicated at this stage is one which shall improve digestion. The whole gamut of the materia medica has been run for such a remedy. Tonics, alteratives, sedatives, stimulants, etc., have in turn been tried and found wanting.

An analysis of Extract of Malt suggests this article as a valuable agent, and its employment quite justifies the hope inspired of such analysis. Besides its tonic properties, it is rich in food (glucose) which has undergone an important step in the process of indigestion. In the latter property it possesses a great advantage over cod-liver oil, which so often not only offends the impaired stomach, but which is, under most circumstances, so difficult of digestion.

It is further valuable because of its property of emulsifying cod-liver oil, and thus aiding in the digestion of the latter, and, indeed, it is in such combination that I have had most experience in its use. In several cases in which I regarded the oil as indicated it positively disagreed, but became perfectly tolerable when given in emulsion with the malt extract.

In support of the proposition that Extract of Malt merits a thorough trial at the hands of the profession, I submit the following reports from cases which have been under my charge:

Mrs. R. consulted me three years ago last May for an annoying cough. I ascertained that her health had been failing for some time and that she had lost flesh appreciably. She complained of dyspnœa on the slightest exertion. Expectoration was very scanty and of tenacious mucus, with occasional streaks of blood. Digestion very imperfect, with much acidity and eructation. Appetite very poor. Inspection revealed diminished expansion in the infra-clavicular portion of left lung. Percussion elicited but slight dullness. Auscultation showed respiration to be interrupted in its rhythm and somewhat bronchial. Here was a case of incipient fibrous phthisis, a diagnosis made more clear by its subsequent history. I placed her on cod-liver oil, and ordered a mixture for the relief of the annoying cough. The latter had the effect of giving slight relief, but the oil was not tolerated. After three months it was painfully manifest that the disease was progressing.

Shortly after this she became pregnant, and this was followed by an improvement of all the symptoms referrable to the lungs. With the exception of a slight roughness in the respiratory murmur, no trace of the lung difficulty romained at the sixth month of pregnancy. At the seventh month, however, she miscarried; the child lived thirteen hours Immediately after the unfavorable lung symptoms returned with increased severity, and continued in spite of treatment. I then placed her on Extract of Malt. After taking two pints of the plain extract, an improvement in her digestion and in her general symptoms was noticeable. I then gave her the combination of the extract with cod-liver oil. After a bottle of this, (which agreed perfectly) had been taken she was able to leave her bed for the first time since her confinement, three months previous. She has since been taking regularly the malt with the oil in combination, and on a visit to her yesterday I found that the disease had made no progress since my last examination some three months ago. The respiration sounds have improved, if anything, and I am led to hope that ultimate permanent good may follow the treatment. I cannot but believe that had the Extract of Malt been resorted to when I was first consulted a train of very threatening symptoms might have been averted.

Miss W., aged 19, consulted me October 5, 1877. Her's was a typical case of chlorosis. She had consulted and had been treated by several of our leading physicians, but without benefit. Supposing that the usual remedies in such cases had been employed, I placed her on Extract of Malt with cod-liver oil and iodide of iron. The improvement which followed was very marked, and after the sixth pint had been taken, menstruation, which had been suspended for several months, was re-established, and after taking two more pints further medication was considered unnecessary.

I regard the combination of Extract of Malt with pepsin as one of the most valuable additions to our list of physiological medicines, and, without taking up your time with the details of cases in which I have employed it, I know of no remedy of such wide application and of such service in functional dyspepsia. In the deplorable condition of the stomach following the drunkard's prolonged debauch, Extract of Malt with pepsin is of great benefit. In the emaciation and condition of marasmus, following the summer diarrhœa of children, it is also a sovereign remedy. The hints on epidermic medication in a recent number of the Michigan *Medical News* by Dr. Wade, of Holly, led me to apply it in the manner recommended and with very favorable results.

Extract of Malt is by no means a specific remedy, and though I have employed it in tuberculosis with little or no benefit, yet I regard it as inferior to no other agent in such cases, while its combinations constitute the most valuable means in our hands.—*Mich. Med. News.*

### PROGNOSIS IN CEREBRAL HEMORRHAGE.

It is often important to be able to give a reasonably correct opinion as to the result of apopletic attacks, in answer to inquiries by friends and parties interested. Dr. Lapponi, in the *Revista Clinica de Bologna*, presents some valuable hints on this subject, which may be epitomized as follows:

Those attacks in which coma continues over twenty-four hours are fatal. There are a few exceptions which extend the farthest limit to three days.

There are but few attacks followed by slightly prolonged coma, in which one fails to observe, before the return of consciousness, occasional yawnings separated by intervals more or less prolonged. But fi these yawnings occur soon after the attack, if they are frequent and succeed each other rapidly, a fatal termination is certain.

Paralysis of the buccinator always indicates a serious attack, as the seat of lesion is not far from the medulla oblongata. Equally grave, and perhaps more so, is labio-glosso laryngeal paralysis, which the author thinks he was the first to observe. Here the paralysis is of the hypoglossal and a portion of the facial nerve, from lesion of bulb.

All cases in which, thirty or forty minutes after the attack, vomiting occurs without nausea or effort, being a veritable regurgitation of the contents of the stomach, will terminate in death. The value of this symptom is due to leision of the vagus nerve.

Paralysis of the pharynx, from leison of the origin of the vagus, and polyuria supervening a few hours after the attack and due to lesion of the bulb, alike indicate great danger.

Extreme depression of temperature occurring soon after the attack, is often the prelude of death. But if there succeed to this initial fall of temperature a reaction which raises the temperature above the normal standard, the prognosis is unfavorable without exception.

Finally, the decubitus acutus, so well described by Charcot, is a fatal symptom. —*Pacific Med. Jour.*

### CAFFEINE IN CEPHALALGIA.

Citrate of caffeine, in doses of two grains every half hour until the pain ceases, is strongly advocated as an effectual remedy. Often one or two doses are quite sufficient. The only contra-indication is sleeplessness, which sometimes results if it is taken in the evening. It is preferable to guarana as being hardly ever rejected by the stomach. In hay fever, spinal irritation, and general neuralgia, it would seem worthy of a trial. —(*Montpelier Medical.*)

